# Passive Photonic Integrated Circuits Elements Fabricated on a Silicon Nitride Platform

**DOI:** 10.3390/ma15041398

**Published:** 2022-02-14

**Authors:** Marcin Lelit, Mateusz Słowikowski, Maciej Filipiak, Marcin Juchniewicz, Bartłomiej Stonio, Bartosz Michalak, Krystian Pavłov, Marcin Myśliwiec, Piotr Wiśniewski, Andrzej Kaźmierczak, Krzysztof Anders, Stanisław Stopiński, Romuald B. Beck, Ryszard Piramidowicz

**Affiliations:** 1 Institute of Microelectronics and Optoelectronics, Warsaw University of Technology, Koszykowa 75, 00-662 Warsaw, Poland; mateusz.slowikowski@pw.edu.pl (M.S.); bartlomiej.stonio@pw.edu.pl (B.S.); marcin.mysliwiec@pw.edu.pl (M.M.); piotr.wisniewski@pw.edu.pl (P.W.); andrzej.kazmierczak@pw.edu.pl (A.K.); krzysztof.anders@pw.edu.pl (K.A.); stanislaw.stopinski@pw.edu.pl (S.S.); romuald.beck@pw.edu.pl (R.B.B.); ryszard.piramidowicz@pw.edu.pl (R.P.); 2 Centre for Advanced Materials and Technologies CEZAMAT, Warsaw University of Technology, Poleczki 19, 02-822 Warsaw, Poland; maciej.filipiak@pw.edu.pl (M.F.); marcin.juchniewicz@pw.edu.pl (M.J.); bartosz.michalak@pw.edu.pl (B.M.); krystian.pavlov@pw.edu.pl (K.P.)

**Keywords:** silicon nitride, photonic integrated circuits, silicon photonics, arrayed waveguide grating, generic technology, photonic sensors

## Abstract

The fabrication processes for silicon nitride photonic integrated circuits evolved from microelectronics components technology—basic processes have common roots and can be executed using the same type of equipment. In comparison to that of electronics components, passive photonic structures require fewer manufacturing steps and fabricated elements have larger critical dimensions. In this work, we present and discuss our first results on design and development of fundamental building blocks for silicon nitride integrated photonic platform. The scope of the work covers the full design and manufacturing chain, from numerical simulations of optical elements, design, and fabrication of the test structures to optical characterization and analysis the results. In particular, technological processes were developed and evaluated for fabrication of the waveguides (WGs), multimode interferometers (MMIs), and arrayed waveguide gratings (AWGs), which confirmed the potential of the technology and correctness of the proposed approach.

## 1. Introduction

In the last two decades, photonic integrated circuits (PICs) attracted significant research interest and took an increasingly stronger position in the market, especially in the fields of telecom, datacom [1,2], and sensing applications [3,4]. This is due to the numerous advantages of PICs: miniaturization, high reliability, energy efficiency, and reduction in manufacturing and packaging costs. Among several technological platforms dedicated to integrated photonics, two have the dominant position on the market: silicon-on-insulator (SOI) and indium phosphide (InP), with recently increasing importance of silicon nitride (SiN) platform [5]. The key difference between silicon-based and indium phosphide technologies is the possibility of fabricating active photonic elements. Unlike silicon platforms, indium phosphide (InP) platform allows monolithic integration of active components like semiconductor optical amplifiers (SOAs) and lasers, which is a consequence of the direct energy bandgap of InGaAsP forming the active layer [6]. What is more, by manipulating the composition of InGaAsP, bandgap engineering is also possible, which offers the freedom of shaping the spectral range of operation while keeping the lattice constant matched with the InP substrate (in general, the range 1100–1600 nm is technologically available on the InP-based platform) [7,8,9,10].

Si-based platforms (SOI and SiN), based on mature technology, offer the advantages of low loss elements and tighter monolithic integration of passive components, compatibility with standard CMOS technology, and thus, the natural potential for integration with integrated electronic circuits. Both platforms suffer, however, from the indirect bandgap of the silicon which makes monolithic integration of the light sources and amplifiers impossible. Despite the technological similarities [11] a fundamental differences can be also determined for silicon and silicon nitride platforms [12]. The first significant distinction is the spectral range. For silicon PICs, low absorption losses start for wavelengths longer than 1100 nm, while for silicon nitride low loss range starts from 400 nm [13], which enables shifting the operational spectral range of PICs to the visible part of the spectrum, thus extending the application area [10,14]. A second significant difference is the refractive index value, which for silicon is c.a. 3.5, while for silicon nitride c.a. 2.0. Both platforms use silicon oxide with a refractive index of c.a. 1.5 as a cladding. A large refractive index contrast, characteristic for the silicon platform, allows obtaining small footprint of elements and make chips more compact. A minimum bending radius of 100 μm is feasible in silicon nitride platform, based on the criterion that bend losses should be below 0.01 dB/cm for 1.55 μm wavelength [15]. For silicon on insulator platform bending radius can be as small as 5 μm without an impact to propagation loss [16]. Single mode waveguides made of silicon nitride [7] have larger cross-sections than those made using silicon on insulator (SOI) platform [7,16]. This allows using less sophisticated and cheaper technological processes for their manufacturing. Common features of silicon and silicon nitride platforms are advanced, mature, and reliable technology based on CMOS processes and great potential for monolithic integration with driving microelectronics circuits [17].

The integrated photonics technology was already mastered by several key players on the PICs market. Companies like Intel, IBM, Luxtera, A*STAR, GLOBALFOUNDRIES, INPHOTEC, TowerJazz, LioniX, SMART Photonics, LIGENTEC, and Infinera, to enumerate only a few, dispose of a confirmed potential of manufacturing market-ready PICs [18]. Also, several research centers developing integrated photonics technologies can be easily enumerated (e.g., CEA-Leti, IHP Microelectronics, Sandia National Laboratories, IMEC, VTT, HHI, TU/e, AMO, CORNERSTONE). Also, other companies, research centers and universities work on development of their own technological platforms.

In this work we report the full, successful development flow of building blocks of the first Polish photonic integrated SiN-based platform that includes waveguides, multimode interference (MMI) couplers, and the most complex element—arrayed waveguide gratings (AWGs). We present the main simulation, design and manufacturing steps, present and comment on the results of the optical characterization of developed structures, and discuss major challenges with respect to future work. The visible spectral range, potentially interesting for bio-photonic applications, was chosen for this proof-of-the-concept research work, however, the platform might also enable reaching IR and mid-IR spectral ranges in the future.

## 2. Materials and Methods

### 2.1. Simulations and Design

A development flow of photonic devices typically starts with a definition of an acceptable value range of their performance parameters. Then, analytical calculations can be performed based on those values to obtain first estimate of the device geometrical dimensions. Numerical simulations are the next step with the purpose to finetune the device with respect to its compactness, low-loss operation, or meeting other specific requirements. At each step, design parameters have to be cross-checked with manufacturing constraints. Commercial software packages are available on the marked aiding development flow and were used for numerical simulations and devices GDS extraction presented in this work. PhotonDesign FIMMWAVE, FIMMPROP, and EPIPROP packages were utilized [19].

Apart from analytical calculations, two numerical computing methods were utilized: Finite Difference Method (FDM) and Film Mode-Matching Method (FMM). FDM approximates differential equations with difference equations over a mesh. Reduction of differential equations to algebraic ones that are better suited for modern computers architectures makes this method a powerful tool for numerical analysis. Film Mode-Matching method (FMM) was implemented in revised Sudbo’s formulation [20] and is a semianalytical, fully vectorial waveguide solver dedicated to finding modes in rectangular waveguides [21].

**Figure 1:**
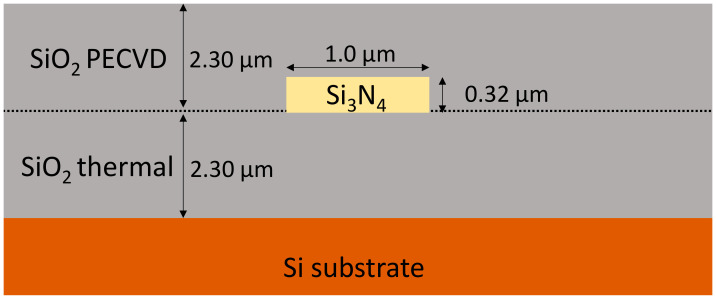
A cross-section of Si_3_N_4_ waveguide.

Based on initial simulation results, it was determined that the presence of SiO_2_ cladding is essential for low-loss propagation and sufficiently high confinement factor. SiO_2_ cladding provides symmetry of refractive index distribution around the waveguide, and hence, symmetrizes the distribution of the mode field. The modes propagating in structure without SiO_2_ cladding tend to leak into the bottom layer of SiO_2_ due to its higher refractive index compared to air. Therefore, all of the structures reported in this work were designed as symmetric waveguides with 2.3 μm thick top and bottom SiO_2_ cladding. A cross-section of the exemplary waveguide is presented in Figure 1.

Si_3_N_4_-based structures performance was examined in numerical simulations. The first set of elements was designed afterwards. WGs, MMIs, and AWGs were optimized for wavelengths: 380, 470, 550, 590, 610, and 660 nm to cover wide wavelength range in VIS passband. Waveguides height was set to 0.32 µm that provides satisfying mode profile and WGs cross-section within typical values for existing nitride-based platforms [10,22].

#### 2.1.1. Straight and Bend WGs Geometries

WGs having the width of 1.0 µm and height of 0.32 µm were chosen for connecting the MMIs and AWGs on chip. This WG geometry is a compromise between the quality of the manufacturing process for silicon nitride structures available in the foundry at the time of manufacturing, number of modes supported by the waveguide, and edge-coupling efficiency. Following the performed simulations, single-mode WG operation for height of 0.32 µm would require width as small as 0.3 µm, which would pose risk of low yield and high WG propagation losses. Furthermore, for sensing applications, multimode devices are sufficient as majority of optical power propagates in fundamental mode. Results of guided modes simulations for WG of such design are presented in Table 1. For waveguide bends, simulations proved, that radiuses as small as 20 µm would support guided modes, however, R = 100 µm were chosen as minimal to keep the bends resistant to potential fabrication imperfections. For WGs with width equal to 1.0 µm and bending radius of 100 µm, there are eight guided modes present while operating at 660 nm wavelength. For these conditions, numerical simulations indicate relative low loss propagation below 2 × 10^−7^ 1/cm (8.7 × 10^−6^ dB/cm) for all modes. Simulations were carried out for an ideal waveguide, origins of the losses induced in physical structures and measurement results are reported in the following sections of this work. Bend modes were simulated with FDM complex solver and fine-tuned with FMM complex solver both utilizing numerical methods mentioned above.

Results of mode simulations are presented in Table 1 for straight waveguides operation at *λ* = 660 nm. For fundamental TE mode, FWHM equals 0.72 µm and 0.32 µm in X and Y direction, respectively. For fundamental TM mode FWHM equals 0.77 µm and 0.27 µm in X and Y direction, respectively.

**Figure 2:**
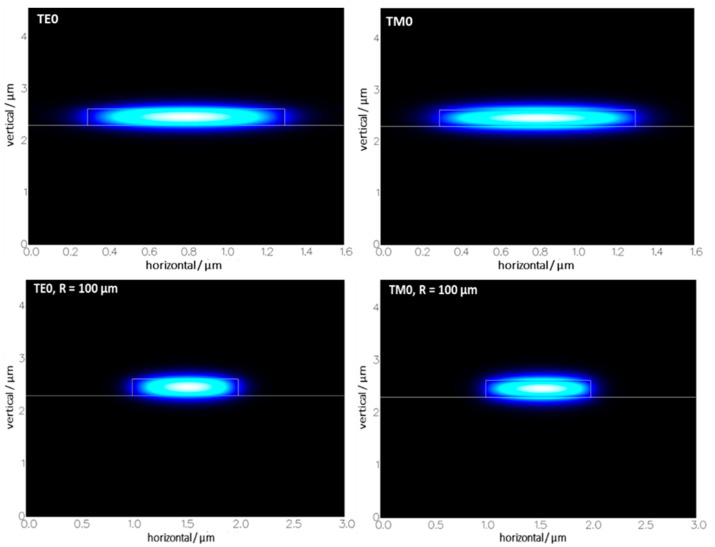
Cross-sections of fundamental modes for straight (**up**) and bend WGs (**down**).

For WG bends with the width of 1.0 µm and bending radius of 100 µm, there are 10 guided modes versus 11 found for straight WGs, and in both cases, most of the optical power is transmitted in the fundamental mode as effective index decreases quickly with increasing mode order. Simulation results of bend modes proved fine confinement of the fundamental mode. Comparison of fundamental modes cross-section for straight and bend (R = 100 µm) WGs is illustrated in Figure 2.

Modes in bend WGs are well confined, with effective mode areas of 0.2534 and 0.3137 µm^2^ for TE0 and TM0 modes, respectively.

#### 2.1.2. MMIs Simulations and Design

**Figure 3:**
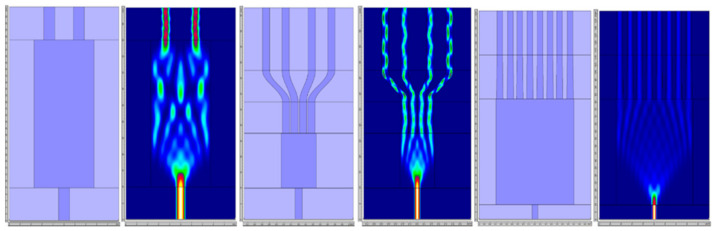
Schematics of simulated 1 × 2, 1 × 4, and 1 × 8 MMI optimized for 660 nm with matched color-maps of total EM-field intensity averaged over *Z*-axis of structures. *X-* and *Y*-axis scales are not matched for better visualization.

We investigated three general types of MMI structures, differing in the number of output ports (two, four, and eight). The investigated types design together with the maps of simulated EM fields are illustrated in Figure 3.

For every MMI, the input WG is nontapered of 1.0 µm width, which results in better field contrast in multimode region (MMR) in respect to devices with tapered input WG. In general, a nontapered input WG results in stronger diffraction at the MMR interface and more confined field maxima in simulation results, which makes the process of shaping and placing the outputs much easier. Output waveguides are straight with the same geometry as input waveguides for 1 × 2 MMIs. For 1 × 4 MMIs, the output section is constructed of three subsections: a 10 µm-long straight section, an s-bend section for more rapid output separation that is closely placed at the end of MMR, and a final straight section. For 1 × 8 MMIs, a two-subsection output is implemented. On the MMR end side, there are 30 µm-long tapers starting with an initial width of 1.2 µm and straight 1.0 µm width section. This approach results in equal power propagating in each output and lower overall losses of the element.

#### 2.1.3. AWGs Simulations and Design

**Figure 4:**
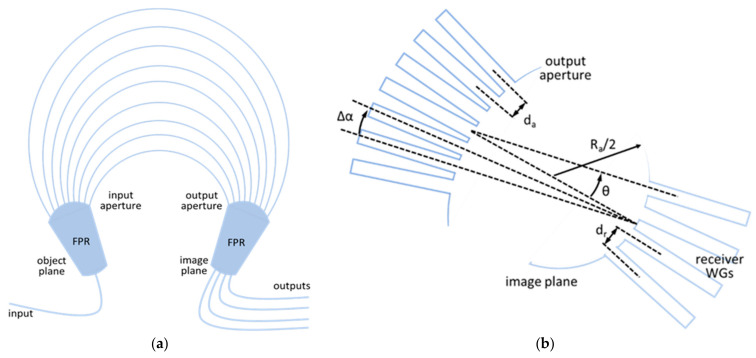
Geometry of AWG: (**a**) layout of arrayed waveguide grating; (**b**) geometry of output star coupler.

To design an AWG layout properly, a number of input parameters are required. The three groups of them can be enumerated: technological parameters determined by the chosen technology process (I/O waveguide width, refractive indices, I/O and array waveguides gaps), type parameters [23] determined by the requirements of the application (number of I/O channels, central wavelength, channel spacing, free spectral range), and transmission characteristics in the form of performance parameters (adjacent waveguides crosstalk, non-uniformity). For predefined layout type, based on the given number of channels, and channel spacing, geometrical design parameters are calculated and presented in Table 2. AWGs have many degrees of freedom; therefore, multiple design strategies can be implemented. Most commonly Smit and van Dam analytical model is used [24] with the recently upgraded model for star couplers [25]. Some standard design flows are well established and widely reported in literature [23,24,26,27]; additionally, design flow aided with novel AWG design software is described in [28]. Most detailed description of design procedure for MUX/DeMUX AWG is given by Smit and van Dam in [23,24]. In this work, a layout consisting of two symmetrical array bend sections comprising WGs of fixed radius connected with straight section WGs is implemented, presented in Figure 4.

The calculated design parameters depend on the specific design approach. An exemplary set of parameters may be as follows: Δ*L*, array order *m*, dispersion *D*, divergence angle of the array waveguides in the array aperture Δα, free propagation region (FPR) radius *R_a_*, length of the arrayed waveguides *L*_i_ and lateral spacing of the arrayed waveguides *d_a_* derived from aforementioned parameters in accordance with the method presented in [27]. This set is defined based on parameters dependencies. AWG characteristics: number of channels *N*, central frequency *f_c_*, channel spacing Δ*f_ch_*, 1-*dB* channel bandwidth Δ*f_L_*, free spectral range Δ*f_FSR_*, central insertion loss *L_0_*, maximum nonuniformity *L_u_*, maximum crosstalk and maximum polarization dependence specify the AWG operation. The list of functional parameters provided by the end-user or defined by the application of the AWG sets the boundaries for accepted values of the design parameters. Set of the maximum acceptable nonuniformity determines maximum dispersion angle and the FPR length which is dependent on the latter [24]. Knowing the limit for receiver spacing and channel spacing, dispersion is also known. WG geometry and material (Si_3_N_4_) determine the minimal phase array (PA) waveguides spacing. For AWGs considered in this work, based on conducted simulations, coupling between neighboring WGs is negligible for spacing larger than 1.25 µm and the smallest distance between the WGs in the array larger than 1.8 µm. Divergence angle and length increment are fixed by choice of parameters discussed above. The width of the input aperture is retrieved from the simulated field distribution on the object plane, and it defines the number of PA waveguides.

Two series of single input AWGs were designed: with four and eight outputs, both optimized for central wavelengths: 380, 470, 550, 590, 610, and 660 nm. Simulation results are presented in Table 2. Rows with structures discussed in detail in following sections have been highlighted.

In addition to design parameters presented in the above table, a detailed description of AWG 100 GHz 1 × 8 optimized for *λ_c_* = 610 nm and 590 nm follows as for those structures the full spectral characterization was conducted.

**Figure 5:**
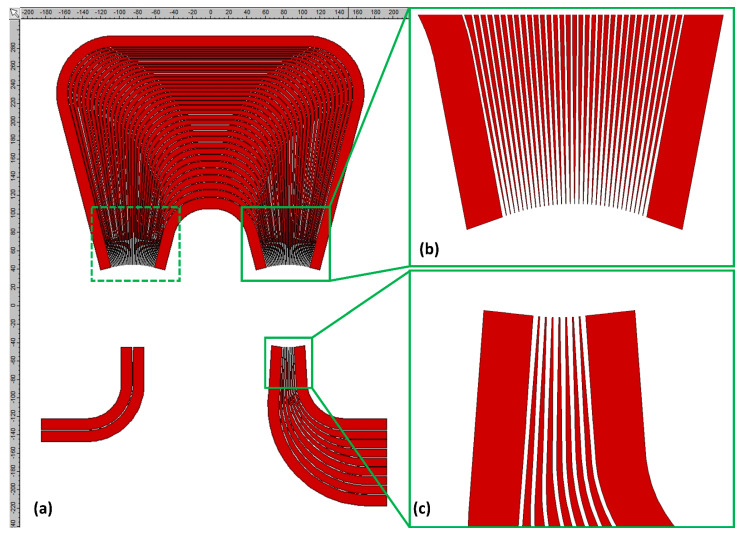
AWG 100 GHz 1 × 8, *λ_c_* = 610 nm simulated layout visualization: (**a**) overall view of AWG; (**b**) I/O section of waveguide array with visible tapers at I/O apertures; (**c**) image plane with output section tapers.

Predefined in Photon Design EPIPPROP Fixed Radius layout was used for all simulations. This layout is symmetric and arrayed waveguides are organized in five sections: straight I/O sections, symmetrical bend sections of equal radius for each of the arrayed WGs and additional straight section for length increment. Bending radius for arrayed WGs was set to 50 µm. This was an acceptable exception from minimal 100 µm bending radius of WGs for routing devices in the PICs. The channel spacing is equal for all AWGs: 0.8 nm (100 GHz). Details of the layout are presented in Figure 5.

For all designed AWGs, taper initial width is a chosen to be standard WG width of 1.0 µm, length 30.0 µm, and endface width is 1.3 µm. The wide etched offset of 12 µm were defined for all layouts to minimize risk of parasitic light coupling in the characterization setup. Array I/O sections are also tapered in input and output apertures in the same manner as input section tapers. Geometry of array I/O tapers differs to take account geometrical differences of generated layouts. Array I/O tapering lowers devices losses. It is, however, a tradeoff between low-loss operation and crosstalk. The same approach was implemented for the output section of tapered WGs at the image plane.

For AWGs 1 × 8 optimized for *λ_c_* = 610 nm and *λ_c_* = 590 nm, simulated phase matching is well preserved in the array. For normalized power injected into the input WG, 0.914 is coupled into the array and 0.799 of the initial power is decoupled at the array end for the first and respectively 0.912/0.753 for the latter.

**Figure 6:**
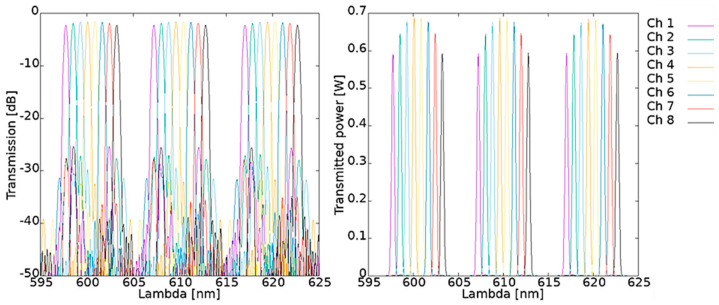
Simulated output spectra of AWG 100 GHz 1 × 8, *λ_c_* = 610 nm.

In Figure 6 the simulated characteristics of spectral response of AWG 100 GHz 1 × 8 optimized for *λ_c_* = 610 nm are presented.

Investigated AWG characterizes with good wavelength robustness with non-uniformity of 0.641 dB and central insertion loss equals 1.640 dB. Average simulated channel spacing is 0.805 nm and FSR equals 9.811 nm.

**Figure 7:**
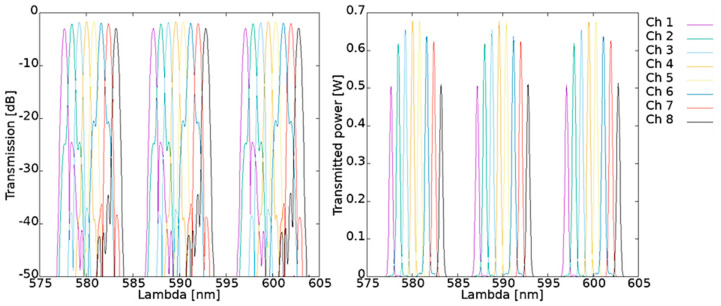
Simulated output spectra of AWG 100 GHz 1 × 8, *λ_c_* = 590 nm.

In Figure 7 simulated spectral characteristics of AWG 100 GHz 1 × 8 optimized for *λ_c_* = 590 nm are presented. This AWG characterizes with non-uniformity of 1.315 dB and central insertion loss equals 1.651 dB. Average simulated channel spacing is 0.805 nm and FSR equals 9551 nm.

Comparison of simulation results of two AWGs discussed above shows good uniformity between the devices in means of power efficiency. However, phase error crosstalk is considerably higher for AWG operating at *λ_c_* = 590 nm. Also, non-uniformity value is higher for that device. Wavefront after grating indicates higher crosstalk for AWG operating at *λ_c_* = 610 nm than for the second one. In fact, only phase error crosstalk shows significant difference between devices.

### 2.2. Mask Layouts Design

**Figure 8:**
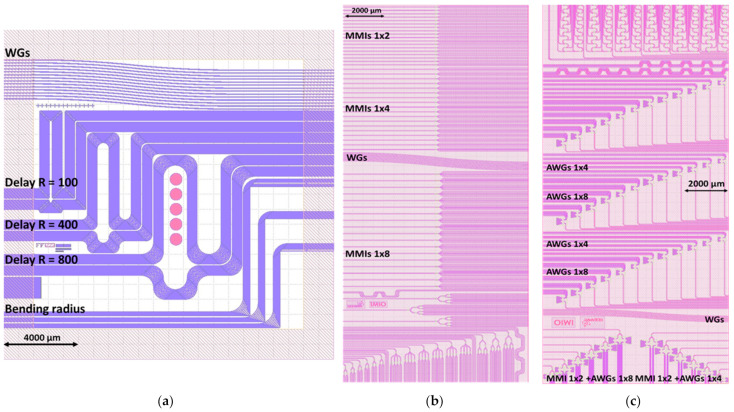
Comparison of layouts: (**a**) Layout 1; (**b**) Layout 2; (**c**) Layout 3.

Based on the results of the simulations discussed in previous subsections, three layouts comprising test structures were designed. The purpose of the first layout was to test WGs geometries. Two following layouts comprised functional devices, connected to the WGs of fixed cross-section (W × H: 0.32 × 1.0 µm). Layouts presented in Figure 8 were designed with an open-source Python-based software Nazca Design environment [29].

The purpose of the first layout was to investigate loss level for different WGS cross-sections. To achieve that goal and gather information on WGs performance, several test structures was designed and placed in the layout. The layout comprises: straight WGs of widths 0.3 µm to 2.9 µm, with 0.2 µm aimed to determine production offset, three series of short and long delay-lines of widths matching straight WGs to determine loss-levels for different cross-sections and bending radii, and series of structures designed to investigate loss on 90° bends. Layout dimensions are 1.8 × 1.8 cm plus 0.4 cm offset for dicing. Structures of each type are multiplied in the layout to provide valid statistical data.

Second layout comprises investigate symmetric MMIs 1 × 2, 1 × 4, and 1 × 8 optimized for 380, 470, 550, 590, 610, and 660 nm wavelength operation. Three copies of each of designed MMI are placed in the layout. This layout also comprises test semi-straight WGs and bend WGs with one and nine 90° bends for reference. Three types of MMIs cascades were designed: first, a simple four-output two-level symmetrical cascade of three MMIs 1 × 2; second, a combined eight-output symmetrical cascade of one MMI 1 × 4 at the first level and four MMIs 1 × 2 at the second level; and finally, a third eight-output three-level cascade comprising seven MMIs 1 × 2. Layout dimensions are 0.9 × 2.0 cm with 0.3 cm offset for dicing.

Third layout is the most complex one, designed for investigating AWGs, 90° bends, MMI 1 × 2 cascades and structures combined of MMIs 1 × 2 and AWGs. Middle section of the layout comprises three series of 12 AWG. Single series consist of six four-channel AWGs and six eight-channel AWGs, one for all central wavelengths of interest. Inputs and outputs of AWGs are placed on opposite edges of the layout with applied offset. Upper section consists of 12 combined structures. Bottom section consists of 90° bend WGs delay lines comprising 32 × 90° bends and 18° bends, five WGs each. There are also diagnostic WGs on the layout. Last structure class are 1 × 2 MMI cascades for investigation of reproducibility of losses induced by a single MMI 1 × 2. Layout dimensions are 0.9 × 2.0 cm with 0.3 cm offset for dicing.

### 2.3. Fabrication Processes

Fabrication starts by precleaning procedure. For this purpose, standard processes were used, identical to those in the CMOS technology, namely, SC-1, SC-2, and Piranha [30], which allow to remove all organic and metallic contaminations from the wafer surface.

After the precleaning process, the wet thermal oxidation process is performed at a temperature of 1200 °C. In this process a 2.3 µm-thick SiO_2_ layer is obtained, intended to separate the silicon substrate from the Si_3_N_4_ guiding layer. Then, silicon nitride layer is deposited. In our experiments the Low-Pressure Chemical Vapor Deposition (LPCVD) method was deployed, with dichlorosilane (H_2_SiCl_2_) and ammonia (NH_3_) used as process gasses. Finally, the layers with a thickness of 320 nm were obtained.

Having these prepared it is necessary to transfer the designed pattern—typically either photolithography or electron beam lithography methods are used. Due to the experimental nature of the work, the electron beam lithography was implemented—time consuming and costly, however resulting in an excellent quality of the defined pattern and offering a high level of flexibility. The positive resist was used to define the pattern, from the most popular family of resists for electron beam lithography, based on polymethyl methacrylate (PMMA) [31]. The PMMA concentrations ranging from 4% to 7% (in anisole) were tested to optimize the process. The resist was deposited using a classical spin-coating technique. Depending on the concentration and the spin speed (between 1000 and 4000 rpm), thickness of the layers varied from 300 nm (for the speed of 2000 rpm and 4% concentration) to 1 μm (1600 rpm and 7%). For the final fabrication 700 nm thick layer of the resist was spin-coated using 6% PMMA at 1550 rpm. A conductive coating needs to be applied during an exposure of nonconductive substrates, such as an Si wafer with SiO_2_ and Si_3_N_4_ layers, to an electron beam. In this work, polyaniline-derivative polymer was used to avoid charge accumulation on the surface [32]. A standard layer with a thickness of 40 nm was spin-coated on the resist at 4000 rpm. After spin coating the resists were baked at a hotplate at 150 °C and 90 °C, for PMMA and conductive protective coating, respectively, to evaporate the solvent before the lithography [33].

**Figure 9:**
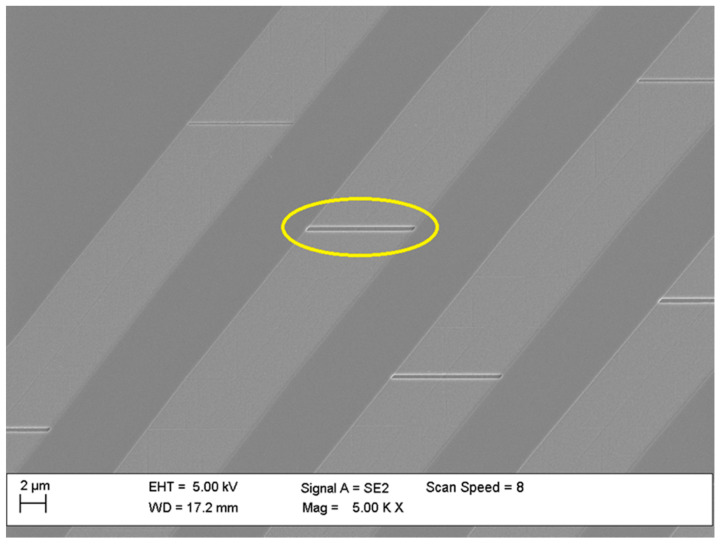
Etched waveguides with marked places of too low an exposure dose during electron beam lithography process.

For experiments with electron beam lithography a system with a beam current of 50 nA and 32 nm beam diameter spot was used. Experimentally determined exposure doses were in the range of 400–1000 μC/cm^2^, depending on the thickness of the resist layer. Final base dose was set at 600 μC/cm^2^. Figure 9 presents a part of the waveguide pattern etched with a too low exposure dose used; a part of the resist was underexposed and remained on the surface after development and etching.

**Figure 10:**
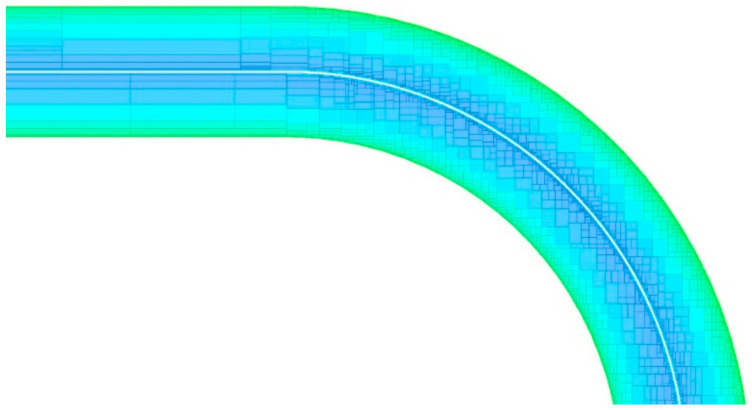
Layout of a waveguide turn with proximity effect correction. Relative dose values are marked with colors—dark blue is lowest dose; green is the highest.

Since PMMA is a positive resist, to manufacture waveguides in the silicon layer, it was necessary to expose to etching the whole surface but waveguiding area. During the preparation of the electron beam lithography process, the proximity effect’s correction was included. This enabled eradication of the effects related to pattern over- or underexposure during the e-beam process due to differences in pattern density. Proximity effect correction involves changing the exposure dose depending on the position of the exposed field in the pattern. The results depend on the sample materials as well as the exposure parameters. In this work, the Monte-Carlo method was used to calculate the proximity effect correction [34]. Due to this solution, exposure dose was precisely defined locally, depending on the changing density of the pattern. The layout of a waveguide bend with the dose correction is presented in Figure 10—blue color indicates areas where the lower relative exposure dose is needed (denser pattern), while the green color indicates higher doses.

**Figure 11:**
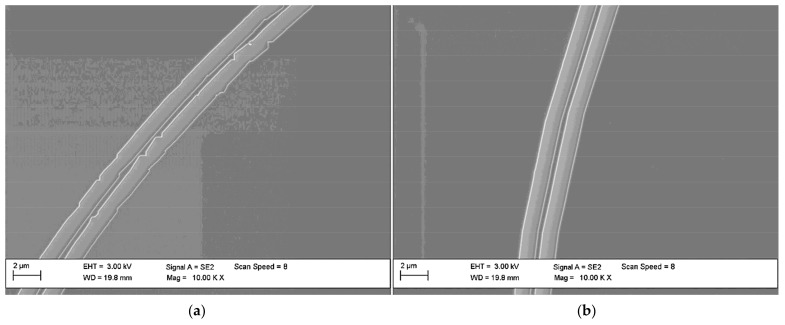
Pattern fracturing: (**a**) before optimization; (**b**) after optimization.

A separate optimization was done for the patterns divided into the exposure fields—fracturing. The exposed pattern is approximated by rectangular exposure fields due to the limitations of electron beam lithography system. While preparing the process, it is possible to choose basic dimensions of rectangles with the limitation of the minimal area size. Figure 11 shows the effect of optimized fracturing.

During optimization, the dimensions and distribution of rectangles was changed, so that they precisely reproduce the curvature of the waveguide in resist.

**Figure 12:**
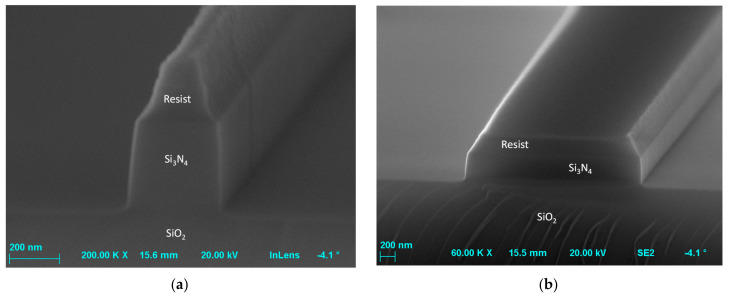
Cross section of exemplary waveguides etched in Si_3_N_4_ with remaining top layer of resist mask of width (**a**) 0.36 µm and (**b**) 2.7 µm.

The developed structures were then subjected to etching process to remove silicon nitride from the exposed areas. The dry etching—reactive ion etching (RIE) with CHF_3_/O_2_ gasses was selected due to its anisotropy, with the 950PMMA resist used as a mask. The determined PMMA: Si_3_N_4_ selectivity was 1.3:1 with an etch rate 31 nm/min. The etched structures were inspected using scanning electron microscope, and the results are shown in Figure 12. Measured wall angle was >86 deg. with low roughness of both walls and bottom of etched structures. Observed defects related to the transferring of layout to Si_3_N_4_ layer, such as a change in dimension and a trapezoidal cross-section, resulting from imperfections of the lithography and etching processes, may result in the later deviation of elements transmission characteristics from simulation results.

After etching, the PMMA mask was removed and 2300 nm layer of SiO_2_ was deposited using PECVD method with process gasses SiH_4_/He and N_2_O.

The final stage of manufacturing of photonic elements is separating the structures. Different methods can be used for this purpose, such as cleaving, dicing, laser ablation, or stealth dicing.

### 2.4. Characterization Setups and Methodology

**Figure 13:**
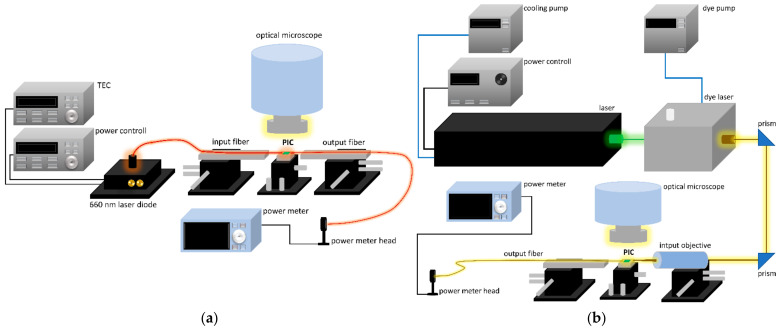
Characterization setups schematics: (**a**) setup utilized for characterization of waveguides (WGs) and MMIs; (**b**) setup utilized for AWGs characterization.

Two measurements setups were assembled for characterization of developed photonic structures. Both are schematically presented in Figure 13.

First (a) setup was used for characterization of operation of WGs, MMIs, and MMIs cascades. The temperature-stabilized red laser diode operating at 660 nm was used as a light source. Light was launched into the chip via optical fiber placed on micrometer translation stage. Also, the photonic circuit was placed on micrometer translation stage and on the output side there was an identical setup as on the input side. Output fiber delivered the light passing through the measured structures to the power meter. No temperature stabilization of the chip was applied apart from the stabilized laboratory temperature. For inspection and easier manual alignment of the setup, an optical microscope was placed above the chip.

Characterization of the AWG was performed using the second (b) measurement setup comprising high-power optically pumped semiconductor OPSL operating at 532 nm with output power 5.16 W, followed by tunable dye laser with Rhodamine G6. The light was coupled into the chip with 50× optical objective. On the output side, the light was coupled into S120C sensor connected via fiber with the power meter. The setup was adjusted manually in the means of the elements position adjustments and wavelength scanning. Measurements were performed for the wavelengths ranging from 570 to 630 nm with an average step of 0.275 nm. Optical spectrometer was used for wavelength control during scan execution and optical spectrum analyzer as more accurate instrument was used for performing reference diagnostic wavelength sweep.

In both setups, chips were mounted on the central stage of the setup with carbon adhesive tape.

## 3. Results

Optical characterization was performed for photonic integrated circuits fabricated in two production runs: 1st run comprising topographies based on initial Layout 1 presented in Figure 8 and 2nd run comprising two following topographies based on Layout 2 and Layout 3 presented in Figure 8. One 4” wafer was processed in each run.

**Figure 14:**
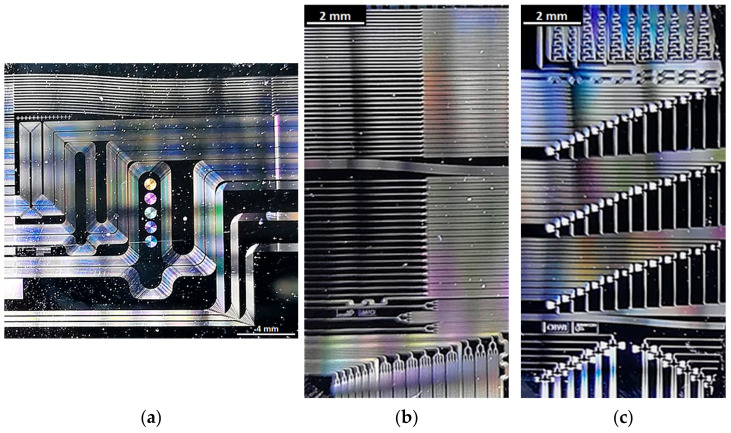
Optical micrographs of fabricated chips: (**a**) 1st Topography (from Layout 1); (**b**) 2nd Topography (from Layout 2); (**c**) 3rd Topography (from Layout 3).

Characterized devices are placed in the chip in series to enable statistics from multiple measurements of the devices of the same type. Each device I/O is routed to the edge interface with WGs. The edge interface was created by manual cleaving with the assist of the table scriber. This method of chip separation was proved to be sufficient for low volume prototyping. Microscopic photographs of exemplary chips of each fabricated type are presented in Figure 14.

### 3.1. Inspection of Manufactured Elements

**Figure 15:**
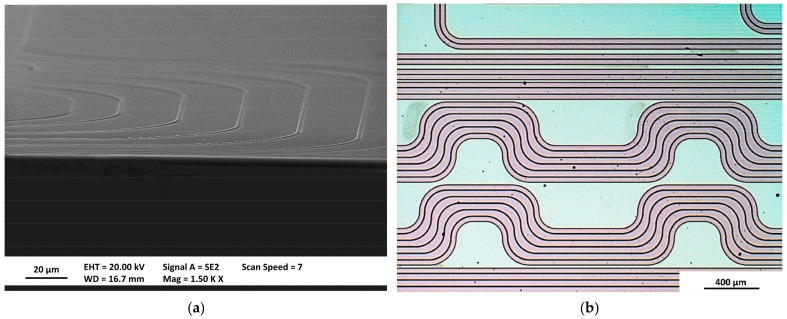
Scanning electron microscopy (SEM) (**a**) and optical (**b**) microscopic images of manufactured WGs.

The visual inspection of manufactured structures was performed with scanning electron microscopy (SEM) and optical microscopes before and after deposition of SiO_2_, before and after wafer dicing. A vast majority of the devices were fabricated accordingly to the design and without any defects. Most promising PICs were selected for characterization. The exemplary structures are presented below. In Figure 15a, there is a SEM image of delay lines. The clearly visible elevations correspond to beneath located WGs. In Figure 15b, there is visual image of the same delay line in which reddish lines mark Si_3_N_4_ etching areas.

**Figure 16:**
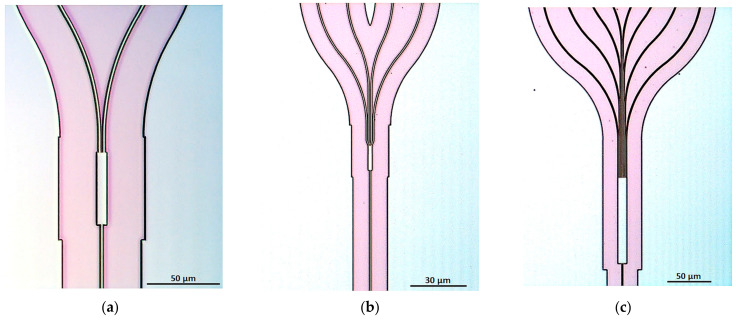
Optical microscope images of MMIs: (**a**) 1 × 2; (**b**) 1 × 4; and (**c**) 1 × 8 optimized for 660 nm operation.

In Figure 16, there are three microscopic images of the MMIs presented: (a) 1 × 2, (b) 1 × 4, (c) 1 × 8, all optimized for 660 nm operation.

**Figure 17:**
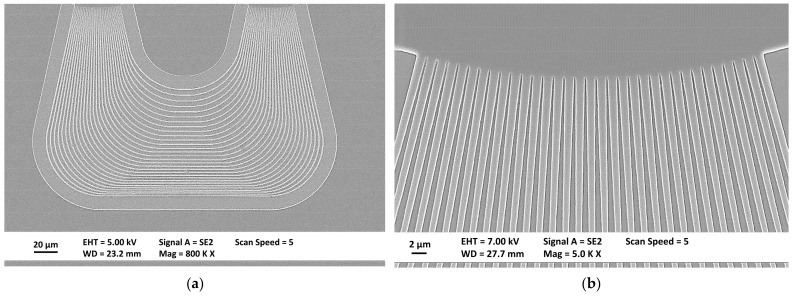
SEM images of etched structures reproduced in resist before etching and final SiO_2_ cladding deposition: (**a**) arrayed WGs of AWG 100 GHz 1 × 4 optimized for 590 nm operation; (**b**) its output aperture magnification.

In Figure 17 there are two SEM images presenting structures reproduced in resist before etching and final SiO_2_ cladding deposition: (a) AWG 1 × 4 phase arrayed WGs; (b) magnification of the PA near output aperture showing correctly etched output tapers; (c) output section of MMI 1 × 2, where right angles of output WGs at the MMR end can be seen; and (d) image plane of AWG 1 × 4, where tapered WGs starting points positions accuracy versus layout can be verified. All four images confirm the correctness of the projection of the layout during fabrication process before SiO_2_ deposition.

**Figure 18:**
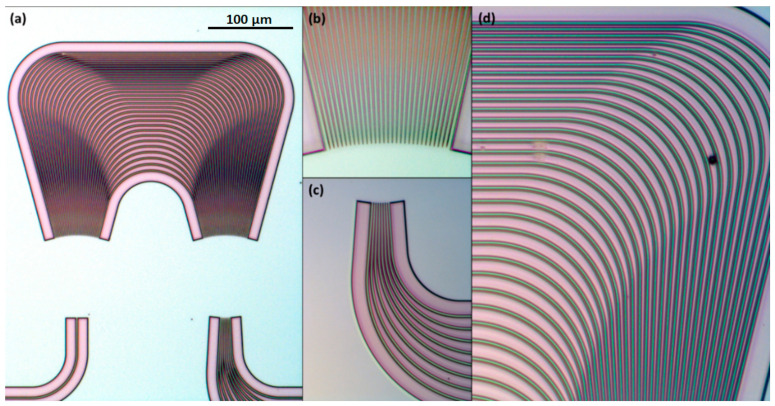
Microscope images of final structures: (**a**) AWG 100 GHz 1 × 8 designed for *λ_c_* = 610 nm; (**b**) a detail output aperture; (**c**) detailed image plane; and (**d**) detailed PA.

In Figure 18 there are optical microscope images presenting a final structure of 100 GHz 1 × 8 AWG designed for *λ_c_* = 610 nm; magnified are the elements crucial for the correct operation of the structure are magnified.

The above-presented micrographs visualize key details of the AWG and confirm the appropriate manufacturing quality.

### 3.2. WGs Characterization Results

To obtain average loss levels of straight WGs, test structures comprising pairs of delay lines of identical cross-sections and length difference of 1 cm were used. The loss originating from additional bends in longer branches of delay lines was taken into account, as 90°-bend loss level was retrieved from separate test structures described in the last paragraph of Section 3.2.

The average optical power losses in WGs were found to be 3.65 dB/cm (*σ* = 1.91 dB/cm) for bend radius R = 100 µm and 1.71 dB/cm (*σ* = 0.50 dB/cm) for R = 400 µm. Each loss level was retrieved as an average of 14 measured WGs on the first chip. The average optical power losses in WGs on the second chip were found to be 3.30 dB/cm (*σ* = 2.43 dB/cm) for bend radius R = 100 µm, also retrieved as an average of 14 measured waveguide pairs. These results show that the WGs performance is well within expected parameters. Obtained values are more than competitive in comparison to commercially available platforms dedicated to visible spectral range [10], which can be considered a significant success. There is, however, a plenty of room for further improvements and better results are expected in the next production runs. A summary of the results for straight WG propagation loss is presented in Table 3.

**Figure 19:**
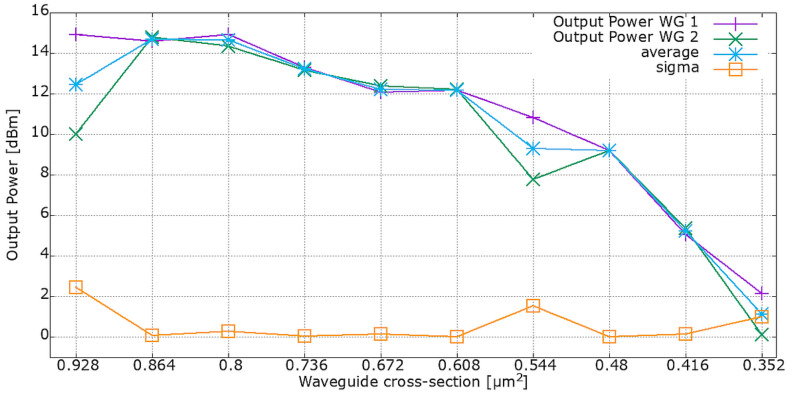
Output power correlation with WG’s cross-sections.

In the 1st chip, WGs of various cross-sections were investigated. The results show an approximately linear correlation in output power with decreasing geometrical cross-section of the WGs. The results are illustrated in Figure 19. Deviations from linear correlation of output power with decreasing WG cross-section can be identified as random defects and differences in coupling quality between particular measurements. There is a negligible standard deviation of the output power for all but three WG cross-sections: 0.928, 0.544, 0.352 µm^2^.

Delay WG lines with 32 and 18 right angle (90°) bends with radius of 100 µm were designed on third layout to investigate single 90° bend loss in standard 1.0 µm width WG based on conclusions derived from the first layout investigation. There are five WGs in each line. The average loss of a single bend equals 0.21 dB (*σ* = 0.01 dB). Small *σ* indicates much better repeatability of manufactured WGs in comparison to first layout-based chip, while the relatively small average loss verifies positively the decision of using tight 100 µm bending radius for the given WG cross-section. A summary of the results for multiple delay lines, five WGs each, is presented in Table 4.

### 3.3. MMIs Characterization Results

Transmission losses of single MMIs could be retrieved from more complex test structures. MMIs cascades with dedicated reference WGs were designed with I/O interfaces on neighboring perpendicular facets on PICs comprising Layout 2 and Layout 3. MMI measurements were conducted using test setup comprising laser diode operating at 660 nm.

First, the reference WGs transmission was measured to determine base transmitted power level and loss on a single 90° bend for the chip comprising MMIs; this was performed as described in Section 3.2. Measurements results for series of five reference WGs with one and nine 90° bends, respectively, indicates the average output power of 9.83 dB (*σ* = 0.13 dB) for a single bend WG, while comparison with nine bends gives average loss of a single bend equal to 0.29 dB (*σ* = 0.01 dB).

**Figure 20:**
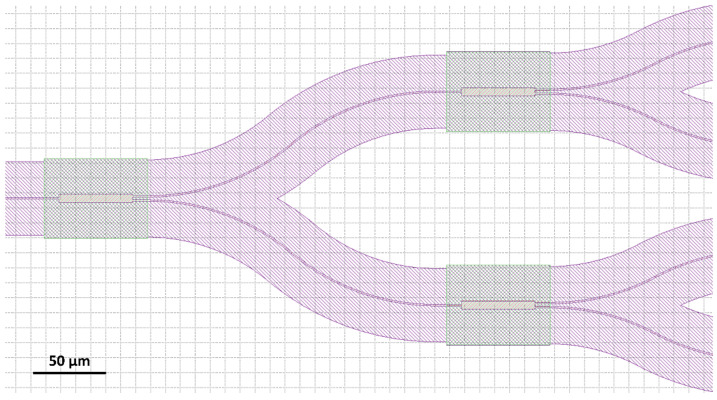
Topography of test structure comprising three 1 × 2 MMIs designed for *λ* = 660 nm.

Performance of 1 × 2 MMIs was investigated by measuring transmission power of a two-level, four-output MMI symmetrical cascade comprising three 1 × 2 MMIs. The layout of such a test structure is presented in Figure 20. Based on these measurements and reference WGs performance on the same PICs, an average loss of single 3-dB MMI splitter designed to operate at 660 nm, measured optical power is, as expected, equally distributed between outputs and the average loss equals 0.49 dB (*σ* = 0.04 dB) for four measurements.

Both the reference WGs and symmetrical cascade comprising three 1 × 2 MMIs measurement results served as the reference for retrieving 1 × 4 MMIs operational parameters. Average loss of 1 × 4 MMI designed for 660 nm wavelength equals 5.53 dB (*σ* = 0.43 dB) for 8 measurements. The origin of exceptionally high measured losses of MMIs 1 × 4 is a geometrical placement of the output tapered WGs. Closely placed outputs resulted in fabrication flaw. Not fully etched narrow trenches in 320 nm SiN layer resulted in change in the length or the multimode region (MMR) of the MMI in respect to design value.

**Figure 21:**
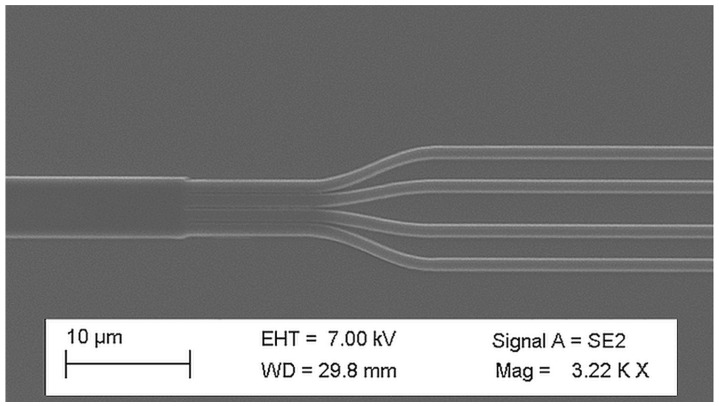
SEM image of 1 × 4 MMI designed for *λ* = 660 nm with visible closely placed output WGs.

For closer illustration of mentioned lossy behavior of 1 × 4, SEM image of fabricated structure is presented in Figure 21. There are two sections visible in the picture. MMR and section of closely placed output WGs is visible.

Additionally, robustness of the MMIs design was tested when 1 × 4 MMI designed for 610 nm wavelength was characterized in the setup equipped with 660 nm laser diode. The average device loss was 9.31 dB (*σ* = 1.55 dB) for 8 measurements. That gives additional 3.79 dB loss while MMI operates under wavelength shifted by 50 nm in respect to the intended wavelength. Loss values were referenced to the power transmitted via diagnostic WGs placed on the chip.

**Figure 22:**
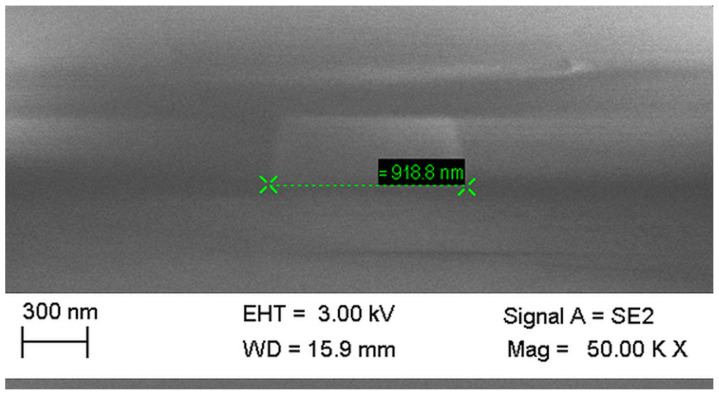
SEM image of cross-section of WG with 1 μm designed width.

The performance parameters of the developed MMIs exhibit very low sensitivity to manufacturing imperfections and input/output waveguides position offsets in respect to devices symmetry axis. Additionally, MMIs show resilience for wavelength offset in relation to the design wavelength. Two main sources of additional losses can be identified: First is mismatch in horizontal dimensions originating in under- or over-etch of the structures and resulting in change of real MMR dimensions in respect to intended ones. In Figure 22, a SEM image of the cross-section of WG with 1 μm designed width is presented. Actual fabricated width is 918.8 μm.

Second source of losses is difference in effective refractive indices between designed and fabricated devices. For 1 × 2 MMIs, FWHM of total transmitted power as a function of wavelength equals approximately 137 nm, meaning that wavelength deviations in range of 75 nm from designed wavelength double the losses of the device in respect to operation at designed wavelength.

### 3.4. AWGs Characterization Results

**Figure 23:**
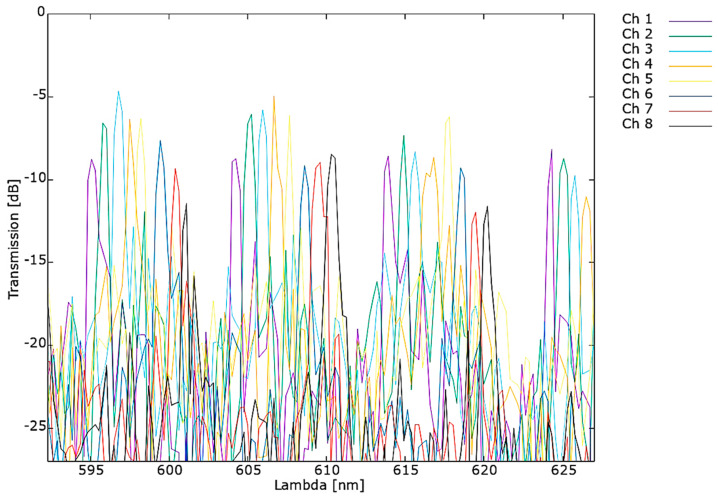
Measured spectrum of AWG 1 × 8 optimized for 610 nm transmission.

The obtained transmission spectra are presented in Figure 23 for 1 × 8 AWG, optimized for 610 nm central wavelength.

The measurements of AWG optimized for 610 nm exhibit a shift of 2.85 nm of the central wavelength in respect to the design parameters. The shift occurs due to the geometrical inconsistence between the designed and fabricated structure mentioned above in Section 2.3. Fabrication processes. Additionally, the difference between structure temperature of 25 °C chosen for simulation and actual temperature of measured structure may also contribute to this effect. The value of the channel spacing equal to 0.79 nm is consistent with the simulations. The free spectral range FSR derived from the measured spectra is 9.23 nm in correspondence to 9.81 nm, resulting from simulations.

**Figure 24:**
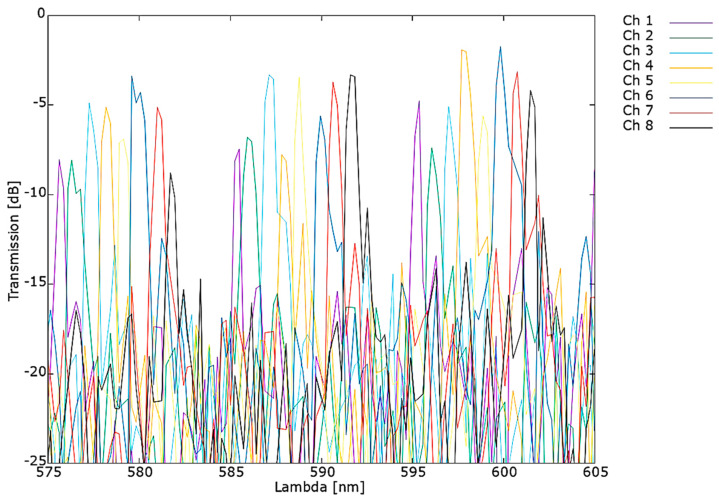
Measured spectrum of AWG 1 × 8 optimized for 590 nm transmission.

Analogous results obtained for AWG 100 GHz 1 × 8 optimized for *λ_c_* = 590 nm are presented in Figure 24.

For this AWG, 1.67 nm shift in central wavelength was measured in respect to design value. The measured channel spacing equals 0.81 nm is consistent with simulations and FSR derived from measurements equals 10.03 nm in correspondence to 9.51 nm simulated FSR value.

## 4. Discussion

Measurements results show satisfying performance of fabricated elements with large optimization and improvement potential in the following runs. Majority of the measurements of WGs and MMIs provided results comparable to those reported for commercial platforms [10]. Comparison of best obtained results to commercial platforms are presented in Table 5.

Correct operation of all types of developed devices was proved during the measurements—the measured parameters of the most complex passive element, which is AWG, are promising and show potential to match commercially available products in the future. Lowering the losses and better matching the design and actual transmission parameters is a matter of further development. Number of issues were addressed and solved along the process and recommendations were noted for further improvements of measurements reliability and efficiency. To achieve lower loss levels, more accurate material properties will be used in the next simulations. Design will also consider manufacturing capabilities and characterization efficiency.

During technological works, the parameters of individual manufacturing steps were established and tested in several iterations. Then, the necessary steps were compiled into the fabrication flow. Critical areas of technology were defined—precise deposition of the Si_3_N_4_ layer, detailed transferring layout in the material, and repeatable separation of structures. The measurement results enabled determining the needs of processes optimization. Better elements definition in material resulting from the optimization of lithography and etching processes will allow obtaining the transmission characteristics of the elements consistent with the simulations. Depositing in a controlled manner a thinner Si_3_N_4_ layer will allow single-mode components fabrication. Modification of cleaving techniques for the separation of structures will reduce the coupling losses. Works on the preparation of new production steps are also planned, allowing the extension of the element library with interferometers, ring resonators, and Bragg gratings.

Measurements of WGs and MMIs proved accuracy of simulations and correctness of the technological process. Conclusions were noted to further improvement and to act as a starting point for development of optical power splitters and (de)multiplexers. Optimal WG cross-sections and design parameters were chosen for the following work. Successful development of AWGs provides foundation to establishing mature material platform and opens new possibilities for applications of SiN-based photonic integrated circuits.

Obtained results and gained expertise enables future optimization of already investigated BBs and development of new elements (e.g., modulators, ring resonators), including attempts to hybrid integration of active components, such as light sources and detectors.

Based on conclusions from an experimental technology run, far-reaching optimization and standardization of design, manufacturing, and characterization process were implemented. Additional steps such double etch depth, metallization for heaters and suspended structures development were scheduled. Investigation of telecommunication band and mid-IR is planned to verify platform operational band scalability difficulty.

## 5. Conclusions

This article reports the full and successful development flow of building blocks for the first Polish photonic integrated platform that includes the most complex passive photonic integrated element: arrayed waveguide gratings (AWG). Visible spectral range was chosen as a promising niche for commercialization in the market of sensing applications.

Obtained results and gained expertise enable future optimization of already developed BBs and development of new elements (such as modulators, ring resonators, tapers, etc.), including attempts to hybrid integration of active components—light sources and detectors.

Fabrication can be mastered regardless of final application targeting visible, near infrared (NIR), and mid-infrared (MIR)/MIR+ spectral ranges [10,35]. Also, integration with driving electronics via wire and flip-chip bonding is to be addressed together with cross-platform integration, thereby allowing the hybrid integration of light sources and detectors.

## Figures and Tables

**Table 1:** Mode parameters simulations results for straight waveguide width 1.0 µm, a chosen waveguide width for routing of fabricated multimode interferometers (MMIs) and arrayed waveguide gratings (AWGs). Assumed wavelength of operation was 660 nm.

Mode	Modal Index	Group Index	β [1/µm]	Confinement Factor	Effective Mode Area [µm^2^]
TE0	1.8892	2.1748	17.9855	0.9791	0.2542
TM0	1.8471	2.2122	17.5841	0.9320	0.3151
TE1	1.8153	2.2424	17.2817	0.9868	0.2930
TM1	1.7809	2.2670	16.9546	0.9393	0.3260
TE2	1.6913	2.3401	16.1014	0.9790	0.3703
TM2	1.6686	2.3586	15.8852	0.9471	0.3499
TE3	1.5390	2.1441	14.6511	0.7047	0.4778
TE4	1.5319	2.2738	14.5841	0.7886	0.5581
TM3	1.5084	2.4610	14.3598	0.9112	0.4159
TM4	1.4847	1.9050	14.1341	0.3697	0.7418
TE5	1.4648	2.0482	13.9448	0.5346	0.8071

**Table 2:** AWGs’ geometrical design and simulated performance parameters: AWG *N* × *M*—I/O numbers, *λ_c_*—central wavelength; NWG—number of WGs in array; RwlR—Rowland radius; order of array; WgSpac—design WG spacing in array; Plength—physical pathlength of device between transition WG tapers; Olength—optical length of device; Wc—worst coupling between PA WGs; Overlap—between str. mode 1 used for AWG layout with corresponding bend mode; FSR—Free Spectral Range.

AWG *N* × *M*	*λ_c_* [nm]	NWGs	RwlR	Order	WgSpac FPR [µm]	Plength [µm]	Olength [µm]	Wc	Overlap	FSR [nm]
1 × 4	380	26	48	50	1.3	391.44	1119.00	0.011	0.987	6.55
	470	30	46	50	1.4	495.21	1420.02	0.008	0.996	8.23
	550	33	46	50	1.5	596.24	797.92	0.380	0.995	9.66
	590	34	45	52	1.4	679.76	555.99	0.055	0.997	9.80
	610	34	45	54	1.3	728.85	1281.37	0.053	0.997	9.86
	660	36	50	52	1.5	786.56	2462.64	0.063	0.996	11.01
1 × 8	380	26	48	50	1.3	491.63	1131.70	0.038	0.987	6.32
	470	30	46	50	1.4	490.22	1232.69	0.012	0.994	8.03
	550	33	46	50	1.5	595.43	1208.68	0.049	0.998	9.36
	**590**	**34**	**45**	**52**	**1.4**	**679.77**	**1176.35**	**0.421**	**0.996**	**9.80**
	**610**	**34**	**45**	**54**	**1.3**	**727.00**	**786.00**	**0.089**	**0.998**	**9.80**
	660	36	45	52	1.5	786.55	1349.48	0.061	0.998	10.71

**Table 3:** Summary of results for straight WG loss, W × H = 1000 × 320 μm, *λ* = 660 nm.

Chip	Bend Radius [µm]	Loss	Number of Measurements	Standard Deviation
1st	100	3.65 dB/cm	14	1.91 dB/cm
**1st**	**400**	**1.71 dB/cm**	**14**	**0.50 dB/cm**
2nd	100	3.30 dB/cm	14	2.43 dB/cm
3rd	100	0.21 dB	5	0.01 dB

**Table 4:** Summary of results for bend WG loss, W × H = 1000 × 320 nm, *λ* = 660 nm.

Delay Line	Av. 32 90° Bends Output Power [dBm]	Av. 18 90° Bends Output Power [dBm]	14 90° Bends Difference [dB]	1 90° Bend Loss [dB]
1	4.31	7.16	2.85	0.2036
2	4.19	7.13	2.94	0.2100
3	4.28	7.07	2.79	0.1993
4	4.47	7.35	2.88	0.2057
5	4.28	7.32	3.04	0.2171
Average				0.2071

**Table 5:** Comparison of WGs’ performance in SiN-based platforms for VIS pass-band.

Group	Type	Wavelength [nm]	WG W [nm]	WG H [nm]	Str. WG Loss (dB/cm)
Ghent [13]	multimode	532	600	180	1.25
Aachen [34]	single mode	660	700	100	0.51
**WUT**	**multimode**	**660**	**1000**	**320**	**1.71**
Imec(BioPIX) [14]	-	835	600	150	0.66

## Data Availability

Not applicable.
